# Antifungal activity of almond (*Prunus amygdalus*) hull extracts against clinical isolates of *Candida albicans*

**DOI:** 10.22034/cmm.2024.345248.1545

**Published:** 2024-01-04

**Authors:** Sara Mozaffari, Hamid Morovati, Shima Gharibi, Vajiheh Azimian Zavareh, Rasoul Mohammadi

**Affiliations:** 1 Department of Medical Parasitology and Mycology, School of Medicine, Isfahan University of Medical Sciences, Isfahan, Iran; 2 Department of Parasitology and Mycology, Faculty of Medicine, Tabriz University of Medical Sciences, Tabriz, Iran; 3 Core Research Facilities, Isfahan University of Medical Sciences, Isfahan; 4 Department of Plant and Animal Biology, Faculty of Biological Sciences and Technology, University of Isfahan, Isfahan, Iran; 5 Department of Medical Parasitology and Mycology, School of Medicine, Infectious Diseases and Tropical Medicine Research Center, Isfahan University of Medical Sciences, Isfahan, Iran

**Keywords:** Antifungal susceptibility, Cell cytotoxicity assay, High-performance liquid chromatography, *Candida* species, *Prunus amygdalus*

## Abstract

**Background and Purpose:**

Various attempts have been made to find potent and effective alternatives with natural origin and fewer side effects for the current antifungals. This study aimed to determine the antifungal effects of the Hydroalcoholic Extract (HE) and Lyophilized Extract (LE) of *Prunus amygdalus hulls* on clinical isolates of *Candida albicans*. Moreover, their effects were compared with fluconazole.

**Materials and Methods:**

Following the preparation of botanical compounds, the toxicity, cell viability, and high-performance liquid chromatography (HPLC) of phenolic compounds analyses were assayed. The broth microdilution method was applied to determine the minimum inhibitory concentration (MIC) values of fluconazole, LEs, and HEs against clinical isolates of *C. albicans*.

**Results:**

According to the HPLC results, the HEs and LEs comprised the main nine components, of which chlorogenic and tannic acids were the most abundant ones. Results of the toxicity assays revealed that no dilution of the extract was toxic to the cells, and the percentage of cell viability was similar to that of the control and above 90% in all dilutions. All isolates showed susceptibility to fluconazole (MIC range: 0.12-1 μg/mL). The MIC geometric mean values of *C. albicans* isolates were 0.29, 11.47, and 48.50 μg/mL for fluconazole, LE, and HE, respectively.

**Conclusion:**

Due to their insignificant side effects and cost-effectiveness, these extracts can be introduced as effective antifungals. Further *in vivo* studies and clinical trials should support the study results.

## Introduction

Candidiasis is a common opportunistic fungal infection caused by *Candida* yeast species. *Candida albicans* is responsible for 50-90% of candidiasis cases [ 1 ]. *Candida* species commonly are the fourth most prevalent leading cause of hospital-acquired bloodstream infections [ 2 , 3 ]. Moreover, it is estimated that 138 million patients per year are reported for recurrent vulvovaginitis [ 4 ]. Therefore, these infections are considered the leading public health issue.

Amphotericin B and fluconazole are two important antifungals applied for the treatment of candidiasis [ 5 ]. However, in recent years, there have been numerous reports of treatment failure by these antifungals in different clinical cases . Various efforts have been made to find potent and effective alternatives with natural origin and fewer side effects for current antifungals [ 8 ]. Several researchers have shown the antifungal effects of various medicinal and condimental plant extracts, such as *Zataria multiflora Boiss* (Shirazi thyme), *Allium sativum* (garlic), *Boswellia sacra* (frankincense), *Syzygium aromaticum* (clove), and *Cinnamomum verum* (cinnamon) on Candida species . This study aimed to compare the potency of our botanical compounds and routine antifungal agent fluconazole.

*Prunus amygdalus* (almond), from the *Rosaceae* family, is known as a source of nutrients. Central Asia, Spain, Australia, and the United States are the leading producers of *P. amygdalus* worldwide [ 14 , 15 ]. The essential oil and medicinal products obtained from *P. amygdalus* are mainly from its shell, which contains flavonoid and phenolic compounds. *Prunus amygdalus* hulls are a source of flavonoids, phenolic, and betulinic acid compounds [ 16 ]. Its extract has been widely used as food seasoning, beverages, healthcare products, cosmetics, and pharmaceuticals [ 14 , 17 ].

The present investigation was designed to determine the antifungal effects of *P. amygdalus* hull extract against clinical and standard isolates of *Candida species* susceptible to fluconazole.

## Materials and Methods

### Compliance with the ethical standards

All methods were conducted in accordance with the relevant guidelines and regulations. Ethics Committee of Isfahan University of Medical Sciences, Isfahan, Iran supervised and monitored this study in 2016 (Ethical approval code: IR.ARI.MUI.REC.1401.151). Informed consent was obtained from all participants, parents/legally authorized representatives of minors (aged less than 16 years), and representatives of deceased participants involved in the study.

### Samples

In total, 50 clinical specimens, including vaginal swabs, blood, wound, and urinary and gastrointestinal secretions, were collected from patients with confirmed candidiasis who had been admitted to Al-Zahra Hospital in Isfahan, Iran. Samples were cultured on Sabouraud dextrose agar (SDA, Ibresco, Italy) plus chloramphenicol for initial isolation. Previously, polymerase chain reaction-restriction fragment length polymorphism had been applied to identify clinical isolates.

### Plant collection and extraction of Prunus amygdalus hulls

The hydroalcoholic extract (HE) and lyophilized extract (LE) of *P. amygdalus* hulls were applied in the current study. The *P. amygdalus* hulls were collected on September 30, 2022 (Kesheh, Natanz, Iran; humidity: 16%, with an average temperature fluctuating between 2 °C [35.6 °F] and 10.1 °C [50.2 °F]) from 5-year-old trees and dried in the shade at 25 °C. For extraction, 15 g of ground powdered material was added into 300 mL of 80% methanol in a screw cap bottle and put on a rotator at 90 rpm for 24 h. After purification with filter paper (Sterlitech, USA; Cat number: #2050001), some of the extract was dried by a rotary machine. Afterward, 0.009 g of dry extract was dissolved in 1 mL of Dimethylsulfoxide (DMSO) solution to obtain LE, and the rest of the primary extract was applied as a total extract. Finally, the extracts were kept in dark containers at 4 ºC. To determine the total phenol in the extract of *P. amygdalus* hull, Folin–Ciocalteu reagent and spectroscopic method were employed. The phenol content of *P. amygdalus* hull extract was measured as 0.036 mg per g of plant material [ 18 ].

### Cell line and culture conditions

This study obtained human umbilical vein endothelial cells (HUVECs, Pasteur Institute, Iran). The HUVECs (5×105 cells/ml) were grown in High glucose Dulbecco’s modified Eagle’s medium (DMEM; Gibco BRL, UK) appended with 10% fetal bovine serum (FBS), 100 U/mL penicillin, and 100 µg/mL streptomycin (Life Technologies, Germany). The cells were incubated in a humidified 5% CO2 incubator at 37°C. The cells were harvested by Trypsin/ Ethylenediaminetetraacetic acid (EDTA, Invitrogen; Thermo Fisher Scientific).

### Cell viability screening through the MTT dye

For the cell proliferation assay, 3-(4,5- dimethylthiazol-2-yl)- 2,5-diphenyltetrazolium bromide (MTT) dye (Sigma-Aldrich GmbH; USA) was utilized [ 19 ]. In 96-well plates, 100 μL of media was added to each well, and HUVEC cells were seeded at a density of 1×104 cells/well (cells were counted by hemocytometer). Following overnight culture, the cells were subjected to a 24-hour treatment with several dilutions (0, 4, 8, 16, 32, 64, and 100%) of extract in a cell culture medium containing 10% FBS. After the incubation periods, 100 μL of the medium in each well was mixed with 10 μL of MTT stock solution (5 mg/mL), and the plate was incubated for 4 h at 37 °C. The MTT-formazan product was then dissolved by filling each well with 100 µl of DMSO. The microplate reader (Bio-Tek Instruments, USA) was utilized to measure the optical density of the culture media at 570 nm. The formula for the calculation of cell viability was (average absorbance of the intervention group/average absorbance of the control group) × 100. Assays for viability were run in triplicate.

### High-performance liquid chromatography (HPLC) of phenolic compounds

Using 260 and 350 nm detection wavelengths, the *P. amygdalus* hull extracts were filtered (0.22 μm disk, 20 μL), and 20 μL was fed into the Agilent 1090 system (Agilent, USA). The high-performance liquid chromatography (HPLC) was carried out according to instructions provided by Nouraei et al. [ 20 ]. For this analysis, a 250 × 4.6 mm, 5 μm, symmetric C18 column (Waters Crop., USA) was used, along with a 10 × 4.6 mm id, 5 μm guard column. With gradient elution at 25 ºC and a flow rate of 0.8 mL per min, the mobile phase consisted of water, formic acid (99.9:0.1) as solution A, acetonitrile, and formic acid (99.9:0.1) as solution B. The program was as follows: A: B (90:10) held for 1 min, followed by 10-26% B for 40 min, 26-65% B for 30 min, 65-100% B for 5 min followed by equilibration with 0-90% A for 4 min [ 20 ]. The phenolic compounds were identified through a comparison of the retention durations, and the results were expressed in mg per 100 g of dry sample weight.

### Antifungal susceptibility testing

The antifungal susceptibility testing (AFST) was performed according to the Clinical and Laboratory Standards Institute (CLSI) M27-S4 and M27-A3 document recommendations. The *P. amygdalus* hull extracts and fluconazole (Sigma Aldrich, Germany) were diluted in the Roswell Park Memorial Institue (RPMI)-1640 medium (Gibco, UK) buffered to pH 7.0 with L-glutamine and without bicarbonate. Antifungal final concentrations were provided: 0.064–64 μg/mL for fluconazole, 0.064–64 μg/mL for LE, and 2200-2.1 for HE of *P. amygdalus* hulls. *Candida albicans* were cultured on SDA and incubated at 35 °C. The optical density yeast suspension was evaluated by a spectrophotometer at a 530 nm wavelength and transmission of 75-77%. The MIC results were checked and determined visually following 24 h of incubation at 35 °C, by considering > 50% growth inhibition for fluconazole, LE, and HE [ 21 ]. *Candida parapsilosis* (ATCC 22019), *Candida krusei* (ATCC 6258), and *Candida glabrata* (ATCC 15545) strains were used for quality controls (ATCC stands for American Type Culture Collection).

### Statistical analysis

This is a comparative analysis based on the AFST. The SPSS software (version 24.0) was used for statistical analysis of applied central indices (mean, median) and dispersion indices (range, MIC_50_).

## Results

### Results of high-performance liquid chromatography assay

Table 1 illustrates the results of HPLC analyses. According to the results, the HEs and LEs comprised the main nine components, of which chlorogenic and tannic acids were the most abundant ones. The HUVEC cells were exposed to different extract concentrations for 24 h to determine the cytotoxic effects of the extract on cells. 

**Table 1:** Results of high-performance liquid chromatography analyses.

	Retention time	Compound	mg/100gDW
1	26.82	*p*- coumaric acid	2.849
2	28.93	Rutin	0.837
3	30.12	Ferulic acid	1.531
4	14.51	Caffeic acid	8.456
5	14.22	Chlorogenic acid	31.692
6	29.87	Rutin hydrate	0.048
7	5.12	Gallic acid	85.54
8	39.42	Rosmarinic acid	1.323
9	3.19	Tannic acid	28.364

### Results of cell cytotoxicity assay using MTT dye

**Figure 1:**
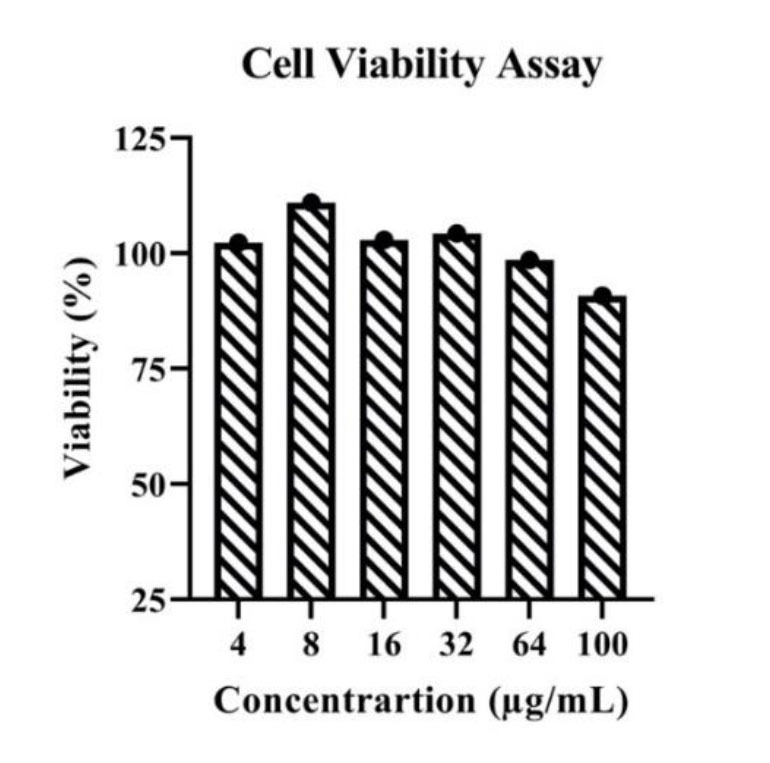
No cytotoxicity of Prunus amygdalus extracts on normal human umbilical vein endothelial cells. Cytotoxic effect of Prunus amygdalus extracts was determined by measuring cell viability using MTT dye after incubation of cells with an increase in concentrations of *P. amygdalus* extracts for 24 h. Data are presented as the mean ± SD and the tests were performed in triplicate.

Results of the toxicity assays revealed that no dilution of the extract was toxic to the cells, and the percentage of cell viability was similar to that of the control and above 90% in all dilutions (Figure 1 and Table 2). Cytotoxic effect of *P. amygdalus* extracts was determined by measuring cell viability using MTT dye after incubation of the cells with increasing concentrations of *P. amygdalus* extracts for 24 h. Data are presented as mean ± SD of the assays that were performed in triplicate (Figure 1).

**Table 2:** Results of the cell viability assay through MTT dye

Sample 1	OD1	OD2	OD3	OD-blank	OD-blank	OD-blank	Mean	SD	Viability (%)	Control	-
Control	0.442	0.453	0.407	0.393	0.404	0.358	0.385	0.02402	100	-	100
100 ug/mL	0.407	0.407	0.382	0.358	0.358	0.333	0.349666	0.014433	90.82	100 ug/mL	90.82251
64 ug/mL	0.438	0.428	0.419	0.389	0.379	0.37	0.379333	0.009504	98.53	64 ug/mL	98.52811
32 ug/mL	0.476	0.43	0.446	0.427	0.381	0.397	0.401666	0.023352	104.33	32 ug/mL	104.329
16 ug/mL	0.43	0.46	0.445	0.381	0.411	0.396	0.396	0.015	102.86	16 ug/mL	102.8571
8 ug/mL	0.489	0.53	0.41	0.44	0.481	0.361	0,4273333	0.060994	111	8 ug/mL	110.9956
4 ug/mL	0.443	0.449	0.436	0.394	0.4	0.387	0.393666	0.006506	102.25	4 ug/mL	102.251

OD: optical density

### Results of antifungal susceptibility testing

Highest and lowest MIC values of fluconazole among *C. albicans* clinical isolates were 1 and 0.12 μg/mL, respectively. Table 3 shows the results of AFST for the studied isolates. Details of MIC values are presented in Table 4. For the HE, the highest MIC of *C. albicans* was 275 μg/mL, and the lowest was 4 μg/mL. Moreover, the highest and lowest MIC values for the LE were 32 and 4 μg/mL, respectively. The MIC_50_ values for fluconazole and LE were 0.25 and 8 μg/mL, respectively. 

**Table 3:** Results of antifungal susceptibility testing of fluconazole and two extract compounds against *Candida albicans*.

Antifungal agents	MIC parameters (μg/mL)					
	Min value	Max value	MIC^50^	MIC^90^	GM	Mean Value
Fluconazole	0.12	1	0.25	0.5	0.29	0.25
HE	4	275	34.5	151.25	48.50	34.3
LE	4	32	8	32	11.47	8

HE: Hydroalcoholic extract, LE: Lyophilized extract, MIC: Minimum inhibitory concentration, GM: Geometric mean

**Table 4:** Minimum inhibitory concentration values of tested antifungals on clinical *Candida albicans* isolates.

No.	Patient Age (years)	Patient gender	Sit of isolation	MIC(μg/μL)		
				Fluconazole	Lyophilized extract	Alcoholic extract
1	21	Female	Esophagus	0.25	8	17.1
2	54	Female	Esophagus	0.25	8	34.3
3	48	Male	Esophagus	0.12	8	34.3
4	37	Male	Esophagus	0.25	4	34.3
5	73	Male	Esophagus	0.12	4	34.3
6	83	Female	Esophagus	0.25	4	17.1
7	43	Female	Esophagus	0.25	8	34.3
8	65	Male	Esophagus	0.25	8	17.1
9	64	Male	Esophagus	0.5	8	17.1
10	57	Female	Esophagus	0.25	8	34.3
11	88	Male	Urine	0.25	8	34.3
12	58	Female	Urine	0.5	8	137.5
13	75	Female	Urine	0.25	8	137.5
14	56	Male	Urine	0.25	16	17.1
15	36	Female	Urine	0.5	16	68.7
16	38	Female	Urine	0.25	8	34.3
17	87	Male	Urine	0.25	16	34.3
18	88	Female	Urine	0.25	8	17.1
19	38	Female	Urine	0.25	8	34.3
20	69	Male	Urine	0.12	8	34.3
21	38	Female	Urine	0.25	16	17.1
22	7	Female	Urine	0.25	8	17.1
23	1 month	Male	Blood	0.25	4	34.3
24	11 days	Male	Blood	0.25	8	34.3
25	74	Male	Blood	1	8	34.3
26	68	Female	Blood	0.25	16	34.3
27	88	Female	Blood	0.5	8	68.7
28	15	Female	Blood	0.25	8	68.7
29	67	Male	Blood	0.25	16	68.7
30	4	Female	Blood	0.25	4	68.7
31	14	Male	Blood	0.5	16	68.7
32	1	Male	Blood	0.5	4	17.1
33	77	Female	Skin	0.25	32	137.5
34	40	Male	Stool	0.25	16	68.7
35	70	Male	Abdominal fluid	0.25	32	275
36	80	Female	Wound	0.25	8	68.7
37	4	Male	Bronchoalveolar lavage fluid	0.5	16	68.7
38	68	Male	Gastric juice	0.25	32	68.7
39	61	Female	Gastric juice	0.25	16	34.3
40	68	Male	Gastric juice	0.25	32	68.7
41	51	Female	Gastric juice	0.25	16	68.7
42	83	Female	Gastric juice	0.25	32	275
43	-	Female	Blood	0.25	16	137.5
44	-	Female	Blood	0.25	32	68.7
45	-	Male	Blood	0.25	32	275
46	-	Female	Vaginal discharge	0.25	32	8.5
47	-	Female	Vaginal discharge	0.5	16	275
48	-	Female	Vaginal discharge	0.5	8	137.5
49	-	Female	Vaginal discharge	1	32	8.5
50	-	Female	Vaginal discharge	0.25	16	275

It means that fluconazole is five times more effective than the LE. Moreover, the MIC^50^ for the HE is 34.3 μg/mL, which is seven times weaker, compared to fluconazole. It was also found that the LE is two times stronger than the HE. Moreover, the MIC^90^ values for fluconazole, LE, and HE were 0.5, 32, and 151.25, respectively. In addition, the exact geometric mean values (GM) for fluconazole, LE, and HE were 0.29, 11.47, and 48.50, respectively. The mean MIC values were 0.25, 8, and 34.3 μg/mL, respectively.

## Discussion

Opportunistic fungal infections are life-threatening health problems in people with immune system defects [ 22 ]. Increase in the consumption of broad-spectrum antibiotics and the rates of immunodeficiencies (HIV/AIDS) and immunosuppressed patients have led to the escalation of systemic fungal infections, mainly caused by *Candida* infections. Due to the high resistance rates and complicated side effects of chemical antifungals, it is required to investigate and develop appropriate treatment tools and methods, mainly based on non-chemicals [ 23 - 25 ].

This study aimed to investigate the antifungal efficacy of lyophilized and hydroalcoholic extracts of *P. amygdalus* against clinical *Candida* isolates. Phenolic and flavonoids of the plant have antifungal and antimicrobial activities. All *C. albicans* isolates were susceptible to fluconazole. Besides, the LE affected 94% of the isolates with a lower MIC than the HE. The GM values of all *C. albicans* isolates were 0.29, 11.47, and 48.50 μg/mL for fluconazole, LE, and HE, respectively.

Geng et al. [ 17 ] studied the antifungal activity of bitter *P. amygdalus* extract against Alternaria and Gloesporium orbicular. Another study by Ibrahim et al. [ 26 ] showed that *P. amygdalus* skin extracts and oil showed potential anti-dermatophyte activities. However, no research was found on the anticandidal effects of the *P. amygdalus* oil. Furthermore, several studies have investigated the antibacterial and antiviral efficacy of *P. amygdalus*. Moreira et al. [ 27 ] showed that *P. amygdalus* skin extract has an inhibitory effect on Escherichia coli, pseudomonas aeruginosa, Listeria monocytogenes, Staphylococcus aureus, and Salmonella. Musarra-Pizzo et al. [ 28 ] showed the antimicrobial and antiviral effects of *P. amygdalus* skin against Staphylococcus aureus and human herpes simplex virus (HSV-1). Mandalari et al. [ 29 ] found that *P. amygdalus* skin has a beneficial impact on balancing colon microbiota and changes the composition of intestinal bacteria, including bifidobacteria and clostridium coccoid in the digestive tract. In another study, they have also reported the potential antimicrobial efficacy of *P. amygdalus* skin [ 30 ]. Bisignano et al. showed the effect of *P. amygdalus* skin polyphenols against Helicobacter pylori and indicated that it can be combined with antibiotics to reduce antibiotic resistance rates [ 31 ]. Abdel-Fattah et al. [ 32 ] suggested that biogenic core-shell nanoparticles in both *P. amygdalus* and berry extracts are effective for cancer treatment and bactericidal aims.

A study [ 33 ] used the disc diffusion method to assay the antibacterial properties of Capparis spinosa on both gram-positive and gram-negative bacteria using ethanolic and petroleum ether extracts. Both extracts demonstrated significant antibacterial effects against Gram-positive bacteria, including Bacillus cereus and Staphylococcus aureus, and also against Gram-negative bacteria, including Pseudomonas aeruginosa and E. coli. Benachour et al. [ 34 ], during an *in vitro* assessment of the antimicrobial activity of Capparis oils against nine bacterial species, showed that the oils lack activity against *E. coli* and display only modest activity against the remaining eight bacterial species. However, the desirability test revealed that the oils utilized were not effective against the bacterial strains subjected to examination.

Kumar et al. [ 35 ] investigated *P. amygdalus* oil against streptozotocin-induced diabetic rats, pm Glut1 protein, and dipeptidyl peptidase IV, showing that *P. amygdalus* significantly reduced blood glucose levels. Dada et al. [ 36 ] investigated the effect of *P. amygdalus* leaves and stem bark on the liver and brain of rats. They concluded that *P. amygdalus* extract can prevent neurological dysfunction and hepatotoxicity caused by cyclosporin-A. Moreover, in another study, Dada et al. [ 37 ] investigated the effect of *P. amygdalus* leaf and stem extract on vital enzymes in the occurrence of high blood pressure during cyclosporine-A consumption. They showed that the extract could be used as a nutritional agent to neutralize heart damage and blood pressure caused by the drug. Chen et al. [ 38 ] found that flavonoids of *P. amygdalus* skin have antioxidant effects and effectively protect LDL against oxidation in hamsters alongside vitamins C and E.

The LEs and HEs of *P. amygdalus* showed an inhibitory effect on the 50 isolates studied in this research. Therefore, these extracts can be introduced as effective antifungals to avoid fungal growth, especially C. albicans fungus. This study aimed to analyze and compare the antifungal potency of *P. amygdalus* extracts and fluconazole against clinical isolates of *Candida* species *in vitro*, which was the main limitation of this study. The *in vivo* design of the study was likely to influence the findings of the current paper.

## Conclusion

The present work focused on the extraction, chemical composition, and antifungal activity of LE and HE of *P. amygdalus*. The HPLC identified the compounds of the extract. Among the nine types of compounds determined by HPLC, the most phenolic compound was gallic acid (85.54), and the most flavonoid compound was Rutin (0.837). The findings suggest that *P. amygdalus* peel extracts inhibit *Candida* species and can be used for antifungal consumption for all tested pathogenic Candida species. The lyophilized extract of *P. amygdalus* skin is more effective against *Candida* species. Based on the results of this work, LE and HE could be developed as antifungals in the future. *In vivo* studies are suggested for further investigations of the antifungal activities of *P. amygdalus* extracts.
